# Selection of Reliable Reference Genes for Gene Expression Studies in the Biofuel Plant *Jatropha curcas* Using Real-Time Quantitative PCR

**DOI:** 10.3390/ijms141224338

**Published:** 2013-12-13

**Authors:** Lu Zhang, Liang-Liang He, Qian-Tang Fu, Zeng-Fu Xu

**Affiliations:** 1 Key Laboratory of Tropical Plant Resource and Sustainable Use, Xishuangbanna Tropical Botanical Garden, Chinese Academy of Sciences, Menglun 666303, Yunnan, China; E-Mails: zhanglu@xtbg.ac.cn (L.Z.); liangl.he08@gmail.com (L.-L.H.); qtfu.xtbg@gmail.com (Q.-T.F.); 2 University of Chinese Academy of Sciences, Beijing 100049, China

**Keywords:** physic nut, reference gene, RT-qPCR, developmental stage, abiotic stress, biofuels

## Abstract

*Jatropha curcas* is a promising renewable feedstock for biodiesel and bio-jet fuel production. To study gene expression in *Jatropha* in different tissues throughout development and under stress conditions, we examined a total of 11 typical candidate reference genes using real-time quantitative polymerase chain reaction (RT-qPCR) analysis, which is widely used for validating transcript levels in gene expression studies. The expression stability of these candidate reference genes was assessed across a total of 20 samples, including various tissues at vegetative and reproductive stages and under desiccation and cold stress treatments. The results obtained using software qBase^PLUS^ showed that the top-ranked reference genes differed across the sample subsets. The combination of *actin*, *GAPDH*, and *EF1α* would be appropriate as a reference panel for normalizing gene expression data across samples at different developmental stages; the combination of *actin*, *GAPDH*, and *TUB5* should be used as a reference panel for normalizing gene expression data across samples under various abiotic stress treatments. With regard to different developmental stages, we recommend the use of *actin* and *TUB8* for normalization at the vegetative stage and *GAPDH* and *EF1α* for normalization at the reproductive stage. For abiotic stress treatments, we recommend the use of *TUB5* and *TUB8* for normalization under desiccation stress and *GAPDH* and *actin* for normalization under cold stress. These results are valuable for future research on gene expression during development or under abiotic stress in Jatropha. To our knowledge, this is the first report on the stability of reference genes in *Jatropha*.

## 1. Introduction

Physic nut (*Jatropha curcas*, hereafter referred to as *Jatropha*) is a perennial, oily seed-bearing plant belonging to the family Euphorbiaceae that most likely originated in Central America and is widely distributed in the tropics and subtropics [1,2]. *Jatropha* has gained attention in tropical and subtropical countries and has spread beyond its center of origin because of its simple propagation, rapid growth, hardiness, drought tolerance, adaptation to wide agro-climatic conditions, high oil content, and multiple uses [3–6].

Recently, *Jatropha* has received much attention as a potential renewable feedstock for producing biodiesel and bio-jet fuel that may replace petroleum in many developed and developing countries [7–9]. However, the full potential of *Jatropha* has not been realized due to several technological and economic factors, one of which is the lack of high-yielding varieties with a high oil content [10–13]. Intensive research efforts have been aimed at improving *Jatropha* growth and yield performance and enhancing beneficial traits, such as a reduced plant height, more natural branching, early maturity, more female flowers, high seed yield with a high oil content, resistance to pests and diseases, drought tolerance/resistance, and an improved composition of lipids suitable for bio-diesel production [14]. Transgenic approaches have the potential to significantly improve the seed productivity of *Jatropha* [15,16] but will require studies of *Jatropha* functional genomics. Because the genome of *Jatropha* has been sequenced [17,18] and genetic transformation technology has been established [19–22], the identification of a large number of agronomically important genes is now in progress.

Quantifying gene expression levels is important for unraveling the function of agronomically important genes. Real-time quantitative polymerase chain reaction (RT-qPCR) is a widely used technique for assessing gene expression and allows the rapid and reliable quantification of transcripts expressed at low levels [23]. However, for the accurate quantification of mRNA transcripts using RT-qPCR, the identification of stable reference genes is crucial for normalizing the levels of target mRNA [24]. Indeed, the use of unvalidated or unstable reference genes may have significant impacts on the results obtained by RT-qPCR and could lead to erroneous conclusions [25–27]. Although housekeeping genes or endogenous control genes that are thought to be unregulated are typically selected as the reference genes for RT-qPCR analysis, several studies have revealed significant variation in the expression levels of these reference genes under different experimental conditions. As no universal reference gene has been identified in plants or animals to date [26], it is therefore pivotal to identify the best potential reference genes for the experimental system under study [28,29].

In recent years, researchers have evaluated the expression stability of reference genes in many species, such as *Arabidopsis* [30–33] and rice [34,35]. A good reference gene is one that is essential for cell function and shows relatively constant expression in different tissues throughout different developmental stages or under different experimental conditions [36,37]. In the last decade, relevant tools for selecting reference genes for RT-qPCR analysis have become available, and several research groups have developed software packages, such as geNorm [38], NormFinder [39] and qBase [40], to identify the most stably expressed genes across a set of samples. These tools are freely available on the web, allowing the identification of the best reference gene for specific experiments. In addition, these programs allow the calculation of a normalization factor over multiple reference genes, which further improve the robustness of normalization. qBase^PLUS^[41] and GenEx [42] are currently considered to be the most powerful, flexible, and user-friendly softwares for RT-qPCR data analyses.

Although there have been many reports describing gene expression analysis under various experimental conditions using different reference genes (*18S* or *actin*) by semi-quantitative RT-PCR [43,44] or RT-qPCR [45–48] in *Jatropha*, a comparison of the different reference genes in *Jatropha* has not yet been reported. Accordingly, we evaluated the expression stability in *Jatropha* of 11 typical candidate reference genes by RT-qPCR among tissues at different developmental stages and under different abiotic stress treatments. Our results reveal that different reference genes should be selected according to the sample type and that a combination of the most stable reference genes provides a more accurate and reliable method of normalization in RT-qPCR analyses.

## 2. Results and Discussion

### 2.1. PCR Amplification Specificity and PCR Efficiency

The melting curves for the amplified products of all 11 candidate reference genes showed a single peak, corresponding to a specific melting temperature (Figure S1a), and agarose gel electrophoresis showed a single band at the correct molecular weight for each product (Figure S1b), indicating good specificity of all the primer pairs used in RT-qPCR. A significant linear relationship (*R*^2^ > 0.99) between the fractional cycle number and the log of the initial copy number was demonstrated for the 11 calibration curves (Table 1). The PCR efficiency of each candidate reference gene, which ranged from 90% for *UBQ-LIKE* to 109% for *UBQ10*, is shown in Table 1, and these efficiencies were used in the calculations for the subsequent expression data.

### 2.2. Transcript Accumulation of Candidate Reference Genes

**Figure 1.**
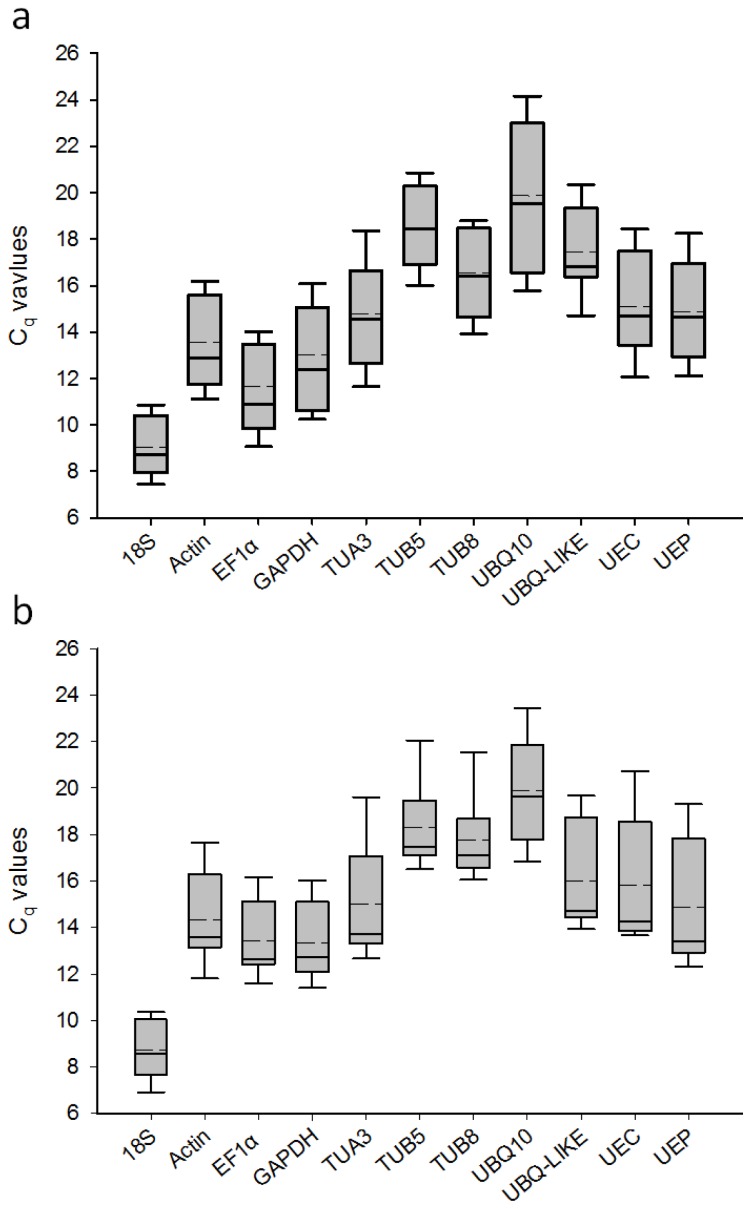
*C_q_* values of the 11 candidate reference genes in the RT-qPCR analysis (**a**) Eleven samples at two developmental stages; (**b**) Nine samples exposed to desiccation and cold stress treatments. The Cq values are shown as the first and third quartile. The vertical lines indicate the range of values. The median values are indicated by solid lines, and the mean values are indicated by broken lines.

The *C_q_* values, which indicate the cycle at which the fluorescence signal is significantly different from background, of the 11 candidate reference genes were obtained by RT-qPCR using two sample subsets. Figure 1a shows the transcript accumulation across 11 tissue samples at two different developmental stages, and Figure 1b shows the transcript accumulation across 9 tissue samples under two different abiotic stress treatments. In both sample subsets, the *18S rRNA* gene always had the lowest C_q_ values, even though the cDNA templates were 50-fold diluted relative to any other genes. In contrast, *UBQ10* consistently showed the highest *C_q_* values. The other three genes with abundant expression levels were *EF1α*, *GAPDH*, and *actin*, with mean *C_q_* values < 15 in all the samples. The average C_q_ values of each candidate reference gene across all the samples and their relative expression variation across all the samples are shown in Supplementary Material (Tables S1 and S2).

### 2.3. Ranking of Candidate Reference Genes and Determination of Optimal Reference Genes

**Figure 2.**
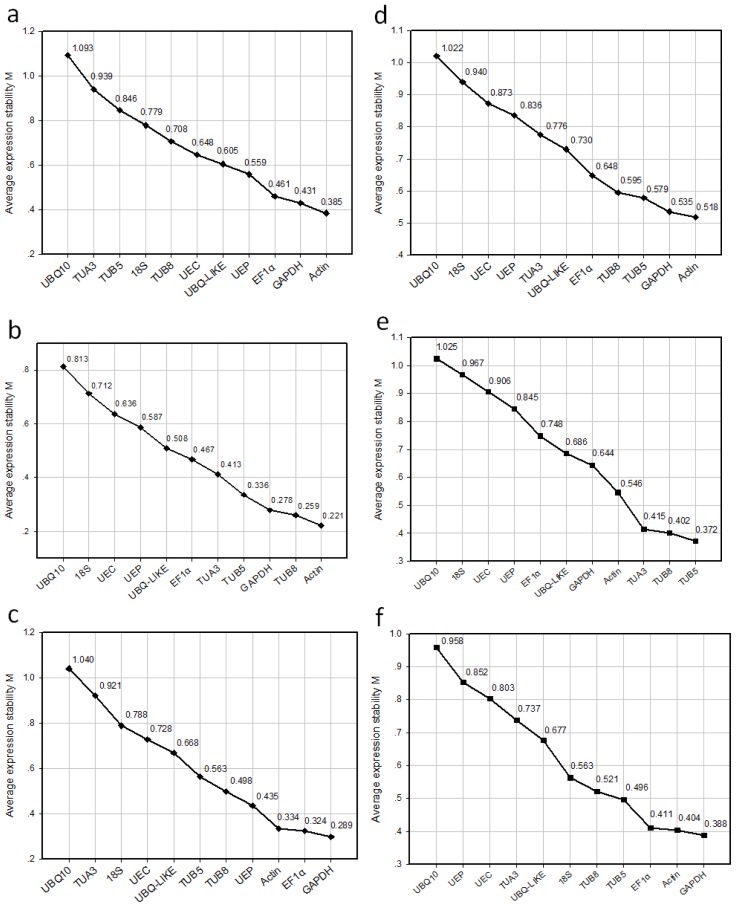
Expression stability values (*M*) and ranking of the candidate reference genes based on qBase^PLUS^ calculations. (**a**) Two different developmental stages; (**b**) Vegetative stage; (**c**) Reproductive stage; (**d**) Desiccation and cold stress treatments; (**e**) Desiccation stress treatment; (**f**) Cold stress treatment. The *M* value and ranking were calculated through a pairwise comparison and stepwise exclusion of the least stable gene. Low *M* values correspond to a high expression stability.

**Figure 3.**
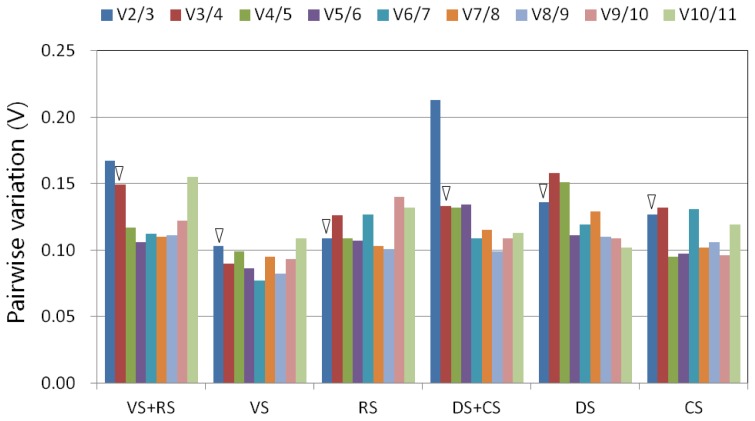
Pairwise variation (*V*) analysis by qBase^PLUS^ to determine the optimal number of reference genes needed for accurate normalization in all the tested samples. *V* values less than 0.15 indicate that no additional genes are needed to calculate a reliable normalization factor. The triangles indicate *V* values lower than 0.15. CS, cold stress; DS, desiccation stress; RS, reproductive stage; VS, vegetative stage.

To minimize the bias generated by the assumptions underlying the different evaluation methods, the expression data in this study were analyzed using two different programs, qBase^PLUS^[40] and NormFinder [39], to rank the candidate reference genes. Based on calculations using the qBase^PLUS^ software, the M values of the candidate reference genes in all six datasets tested were below the default limit of 1.5 (Figure 2). Among the 11 candidate reference genes, the most stable reference genes varied within the sample datasets. In the samples obtained at two different developmental stages, the three most stable transcripts were *actin*, *GAPDH* and *EF1α* (Figure 2a). The *V* value (*V*_2/3_ = 0.167) indicated that the third reference gene, *EF1α*, was required in the developmental series, and the optimal normalization factor can be calculated as the geometric mean of the reference targets *actin*, *GAPDH*, and *EF1α* (*V*_3/4_ = 0.149) (Figure 3). However, the inclusion of only the two most stably expressed genes as references was sufficient in cases in which the datasets contained only one developmental stage, as the addition of a third reference gene did not have a significant effect on the result (Figure 3). At the vegetative stage, the two most stably expressed genes were *actin* and *TUB8* (Figure 2b), and the pairwise variation V_2/3_ was 0.103 (Figure 3), which is below the default cut-off value of 0.15. This result suggests that the combination of *actin* and *TUB8* is optimal for transcript accumulation analysis at this developmental stage. At the reproductive stage, *GAPDH* and *EF1α* were the two most stably expressed genes (Figure 2c), with *V*_2/3_ = 0.109 (Figure 3), indicating that the addition of a third reference gene was also not necessary. Therefore, *GAPDH* and *EF1α* are sufficient for use as reference genes at the reproductive stage.

In the dataset including the samples from the two abiotic stress treatments, the two most stably expressed genes were *actin* and *GAPDH* (Figure 2d), and the pairwise variation *V*_2/3_ value was 0.213 (Figure 3). When a third reference gene (*TUB5*) was added, the pairwise variation (*V*_3/4_ = 0.133) dropped significantly below the default cut-off value of 0.15 (Figure 3). Thus, the normalization factor should preferably contain at least three of the best reference genes (*actin*, *GAPDH*, and *TUB5*). However, the results were different when the samples obtained from the desiccation and cold stress treatments were analyzed separately. The use of two of the most stably expressed reference genes as internal control genes was sufficient for the samples obtained from each of the stress treatments. For the desiccation stress treatment, the combination of *TUB5* and *TUB8* (Figure 2e) was appropriate for gene expression normalization, whereas *GAPDH* and *actin* (Figure 2f) were the best combination for the cold stress treatment (Figure 3). An exclusion analysis was performed to test the possibility that *TUB5* and *TUB8* are co-regulated, whereby each gene was excluded in separate analyses. The ranking of the genes was the same, with very similar M values, indicating a lack of co-regulation between *TUB5* and *TUB8* (data not shown).

In addition to the analysis by qBase^PLUS^, we also evaluated the RT-qPCR data obtained in this study using the NormFinder software [39] and found a similar but not identical ranking of the reference genes in the different sample subsets (Table 2). *Actin*, *EF1α*, and *GAPDH* were found to be the three most stable reference genes in the dataset including samples from two different developmental stages (Table 2), which is consistent with the result determined by qBase^PLUS^ (Figure 2a). In contrast, the ranking of *EF1α* and *GAPDH* differed. In the subset containing only samples from the vegetative or reproductive stage, the two most stably expressed genes determined by NormFinder were *actin* and *GAPDH* or *EF1α* and *UEP*, respectively (Table 2), whereas *actin* and *TUB8* or *GAPDH* and *EF1α* were identified by qBase^PLUS^ (Figure 2b,c).

In the dataset including samples from the two abiotic stress treatments, NormFinder identified *actin*, *GAPDH*, and *UBQ*-like as the three most stable reference genes (Table 2), with *actin* and *GAPDH* also being identified by qBase^PLUS^ (Figure 2d). In the subset containing only samples exposed to desiccation stress, *actin* and *UBQ*-like were identified by NormFinder as the two most stably expressed genes (Table 2), whereas *TUB5* and *TUB8* were identified by qBase^PLUS^ (Figure 2e). However, *GAPDH* and *actin* were identified by both programs as the best combination of reference genes for the subset containing only samples treated with cold stress (Table 2, Figure 2f). Some discrepancies between the rankings obtained from qBase^PLUS^ and NormFinder are expected because the two evaluation approaches are based on distinct statistical algorithms [49].

### 2.4. Validation of the Selected Reference Genes in Leaf Samples Treated with Desiccation or Cold Stress

**Figure 4.**
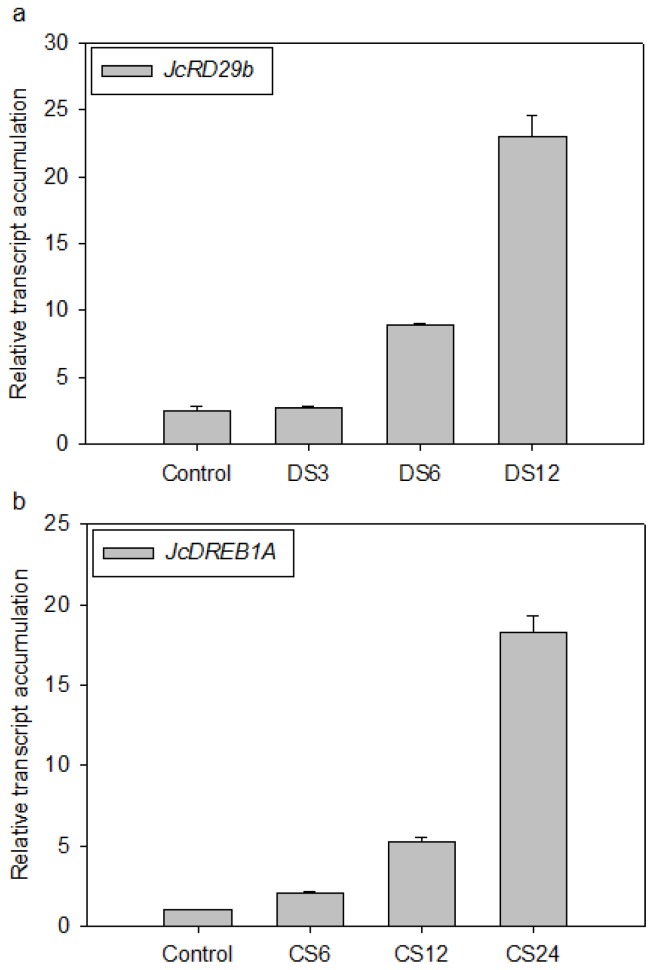

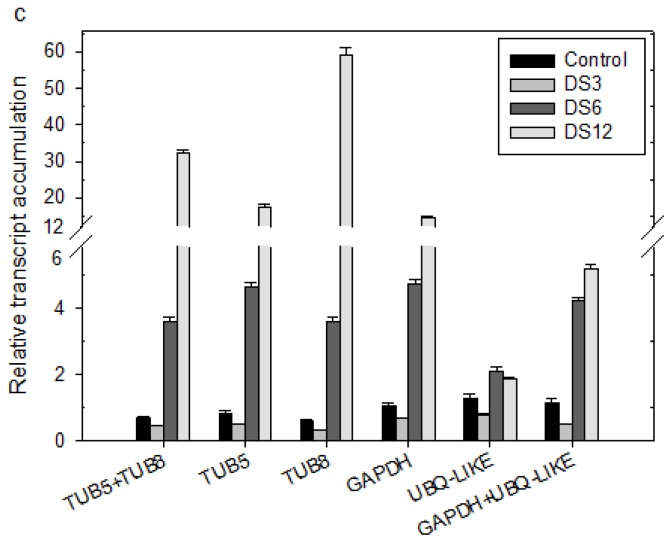
Normalized transcript accumulation of two stress-responsive genes of *Jatropha*, as determined by RT-qPCR analyses. (**a**) Expression profile of *JcRD29b* relative to *TUB5* and *TUB8* in samples exposed to desiccation stress (DS) for 0 (control), 3, 6 and 12 h; (**b**) Expression profile of *JcDREB1A* relative to *GAPDH* and *actin* in samples exposed to cold stress (CS) at 4 °C for 0 (control), 6, 12 and 24 h; (**c**) Comparison of *JcRD29b* expression profiles relative to the two most stable reference genes (*TUB5* and *TUB8*) and the two unstable reference genes (*GAPDH* and *UBQ-LIKE*) in samples exposed to desiccation stress (DS) for 0 (control), 3, 6 and 12 h.

To validate the selected reference genes, the relative expression levels of two stress-responsive genes of *Jatropha*, *JcRD29b* and *JcDREB1A*, were evaluated in leaf samples from plants treated with desiccation or cold stress. The *JcRD29b* and *JcDREB1A Arabidopsis* homologs *RD29b* and *DREB1A* were reported to be induced by desiccation [47] and cold stress [50], respectively. Using the reference genes *TUB5* and *TUB8* (Figure 2e and Figure 3), our RT-qPCR analysis revealed that *JcRD29b* expression was induced at 6 h after the start of desiccation, with its mRNA level significantly increasing after 12 h (Figure 4a). For *JcDREB1A*, the reference genes *GAPDH* and *actin* (Figure 2f and Figure 3) were used to normalize the RT-qPCR data, and we found that the expression of *JcDREB1A* was induced by low-temperature treatment (4 °C) within 6 h, with the strongest expression after 12 h (Figure 4b).

To further validate the suitability of the reference genes identified in this study, we used the two most stable reference genes, *TUB5* and *TUB8*, and the reference genes ranked 5th and 6th by qBase^PLUS^, *GAPDH* and *UBQ-LIKE* (Figure 2e), to quantify the expression profile of *JcRD29b* in *Jatropha* seedlings exposed to desiccation stress. Although normalization using *TUB5*, *TUB8*, or *GAPDH* produced similar expression patterns for each gene, normalization using *UBQ-LIKE* resulted in a strong bias, significantly decreasing the desiccation-induced expression of *JcRD29b* (Figure 4c). This result demonstrates that *UBQ-LIKE* is an unstable reference gene for normalization under conditions of experimental desiccation stress, yet NormFinder defined *UBQ-LIKE* as the second most stable reference gene in the subset containing samples treated with desiccation stress (Table 2). So we conclude that qBase^PLUS^ is preferred for the calculation of normalization factors at least in samples exposed to desiccation stress.

## 3. Experimental Section

### 3.1. Plant Materials and Stress Treatments

At the vegetative stage, tissues (roots, hypocotyls, cotyledons, young leaves, and mature leaves) were collected from *Jatropha* seedlings that were grown in a growth chamber for three weeks after germination (14 h light/day, 20–25 °C). At the reproductive stage, tissues (roots, stems, young leaves, mature leaves, flowers shoots, and seeds at 20 days after pollination [DAP]) were collected from three-year-old adult *Jatropha* plants that were grown in a field at the Xishuangbanna Tropical Botanical Garden (XTBG; 21°54′N, 101°46′E; 580 m elevation) of the Chinese Academy of Sciences located in Mengla County, Yunnan Province, southwest China. All the collected samples were immediately frozen in liquid nitrogen and stored at −80 °C.

The *Jatropha* seedlings that were treated with different abiotic stresses were grown under the same conditions as described above for two weeks after germination (at the three-leaf stage). For the desiccation stress treatment, the seedlings were carefully removed from the soil, and their roots were flushed with tap water. The seedlings were then placed on filter paper and left on the lab bench for the period of time indicated; the roots, hypocotyls, and young leaves were collected at 3, 6 and 12 h after the initiation of the desiccation treatment. For the cold stress treatment, the plants were subjected to 4 °C for 6, 12 and 24 h. Control tissues (roots, hypocotyls, and young leaves) were collected from plants that were untreated. After collection, all the samples were immediately frozen in liquid nitrogen and stored at −80 °C.

### 3.2. Candidate Reference Genes from *Jatropha*

Eleven typical candidate reference genes were selected for analysis in this study (Table 3). The nucleotide sequences of these genes were obtained from the *Jatropha* genomic database [51] or the National Center for Biotechnology Information (NCBI) nucleotide database [52]. The primer pairs used for RT-qPCR were designed using the software Primer Premier 5 and are listed in Table 3.

### 3.3. Desiccation Stress- and Cold Stress-Responsive Genes from *Jatropha*

The *Jatropha* homologs of the two *Arabidopsis* stress-responsive genes *RD29b* [53] and *DREB1A* [50], *JcRD29b* and *JcDREB1A*, were chosen for the RT-qPCR analysis in this study. *Arabidopsis RD29b* is responsive to desiccation and was induced at 3 h after the start of desiccation; its mRNA level reached a maximum at 10 h [53]. The expression of *Arabidopsis DREB1A*, which encodes a dehydration response element-binding protein, was induced within 1 h of exposure to low temperature (4 °C), and the *DREB1A* mRNA level peaked after 2 h [50]. The nucleotide sequences of *JcRD29b* and *JcDREB1A* were obtained from the *Jatropha* genomic database [52]; the primer pairs used in RT-qPCR were designed with the software Primer Premier 5 and are listed in Table 3.

### 3.4. RNA Isolation and Purification and cDNA Synthesis

Two biological replicate samples were collected at each developmental stage and under each abiotic stress treatment. Total RNA was extracted using the silica particle-phenol/chloroform extraction method, as described by Ding *et al.* [54]. All RNA samples were analyzed spectrophotometrically, and their absorbance ratios at 260/280 and 260/230 nm were greater than 1.8. The RNA samples were also analyzed by agarose gel electrophoresis, showing well-defined bands corresponding to each rRNA and no evidence of nucleic acid degradation. The total RNA samples were purified using the RNAclean Kit (TIANGEN Biotech, Beijing, China), treated with DNase I to remove DNA, and immediately reverse transcribed using the PrimeScript™ RT Reagent Kit with gDNA Eraser (Takara Bio. Inc., Dalian, China) according to the manufacturer’s protocol. Either a gene-specific primer mix or oligo-dT primers were used in the reverse transcription reactions. In a preliminary test with three different genes (*actin*, *GAPDH* and *TUB8*), consistent RT-qPCR expression patterns were observed in different tissues (roots, cotyledons, stems, young leaves, and mature leaves), regardless of whether the cDNAs were synthesized using the gene-specific primer mix or oligo-dT primers (Figure S2). Because more transcript accumulation was detected with cDNAs obtained using the gene-specific primer mix (Figure S2), the cDNAs used for subsequent experiments in this study were reverse transcribed with the gene-specific primer mix.

### 3.5. Primer Design and RT-qPCR Analysis

The RT-qPCR primers (Table 3) were designed using the software Primer Premier 5. To verify the sequences of the reference genes, all the amplicons of the 11 candidate genes were analyzed on 1.5% agarose gels and sequenced (Figure S1b and Table S3). Each PCR reaction contained 10 μL SYBR^®^ Premix Ex Taq™ II (Tli RNaseH Plus, Takara Bio. Inc., Dalian, China), 200 nM each primer, and 2 μL diluted cDNA (*18S* cDNA was diluted 50-fold relative to all other genes) in a total volume of 20 μL. No-template controls were also included for each primer pair. The RT-qPCR reactions were performed using the Roche LightCycler 480 Real-time PCR Detection System (Roche Diagnostics, Mannheim, Germany). We chose the annealing temperature with the highest amplification and best product specificity, as determined by a melting curve analysis. The reactions were performed in a 96-well reaction plate under the following conditions: 5 s at 95 °C for the initial denaturation, followed by 40 cycles of 5 s at 95 °C, 20 s at the optimal temperature for each primer pair (Table 1), and 20 s at 72 °C for PCR amplification, and 1 cycle of 15 s at 95 °C, 20 s at 65 °C and 0.06 °C/s heating up to 97 °C for the melting curve. The machine measures the DNA concentration in each cycle when the temperature is at 72 °C. Three technical replicates were analyzed for each biological replicate. The amplification efficiency of each set of primers was tested prior to the expression studies and was calculated as *E* = −1 + 10^(−1/slope)^[55], where the slope is derived from a standard curve generated using four 10-fold serial dilutions of cDNA obtained from young leaves.

### 3.6. Data Analysis

We used a Roche LightCycler 480 Real-time PCR Detection System to collect the fluorescence data. The cycle threshold, *C_q_* (the cycle at which the fluorescence signal is significantly different from the background), was determined for each reaction. The average *C_q_* and standard deviation of *C_q_* for each sample were estimated based on three replicates; outliers were removed from the average *C_q_* calculation. Any replicates showing non-specific products in the dissociation curve analysis were also removed. At least two of the three technical replicates were included in the average *C_q_* calculations.

Two algorithms—qBase^PLUS^ and NormFinder—were used to determine the best reference genes for the normalization of the gene expression data. The software qBase^PLUS^ V2.4 [41], combines proven geNorm and qBase technology, and is thus considered valid software for analyzing reference genes [40]. This method is based on the principle that the expression ratio of two ideal reference genes is identical in all samples, regardless of the cell type or treatment. The pairwise variation of a particular gene from all the other reference genes is calculated and defined as the M value, and the gene with the lowest M value is considered to have the most stable expression. The pairwise variations between two subsequent normalization factors (*V* value, *V_n_*_/_*_n_*_+1_) are also calculated, indicating the effect of including one additional gene for normalization. The recommended cut-off of 0.15 for the *V* value was used in our study [38]. NormFinder uses an analysis of variance (ANOVA)-based model to estimate intra- and inter-group variations and combines these estimates to provide a direct measure of the variation in expression for each gene [39]. The candidates are ranked according to their expression stability.

## 4. Conclusions

This study represents the first attempt to select a set of commonly used candidate reference genes in *Jatropha* for the normalization of gene expression data using RT-qPCR. Our results indicate that more than one reference gene is preferred and that different reference genes should be selected according to the different sample type. According to the results calculated using the qBase^PLUS^ software, the best combinations of reference genes for the normalization of gene expression profiles in *Jatropha* are as follows: *actin* + *GAPDH* + *EF1α* for all developmental stages; *actin* + *TUB8* for the vegetative stage; *GAPDH* + *EF1α* for the reproductive stage; *actin* + *GAPDH* + *TUB5* for desiccation and cold stress treatments; *TUB5* + *TUB8* for desiccation stress treatment; and *GAPDH* + *actin* for cold stress treatment. The results obtained in this study provide information that will ensure more accurate RT-qPCR-based gene expression quantification in *Jatropha*.

## Supplementary Information



## Figures and Tables

**Table 1.** Slopes, amplification efficiency (*E*), correlation coefficient (*R*^2^), annealing temperature (*T_a_*) and melting temperature (*T_m_*) [30] of the selected reference genes.

Gene symbol	Slope	*E* (%)	*R*^2^	*T_a_* (°C)	*T_m_* (°C)
*18S*	−3.21	105%	0.999	58	85.57
*Actin*	−3.299	101%	0.995	58	83.90
*EF1α*	−3.158	107%	0.999	59	82.45
*GAPDH*	−3.173	107%	0.998	58	85.72
*TUA3*	−3.353	99%	0.999	58	86.46
*TUB5*	−3.462	94%	0.999	58	84.05
*TUB8*	−3.285	102%	0.998	58	82.58
*UBQ10*	−3.129	109%	0.998	59	79.79
*UBQ-LIKE*	−3.595	90%	0.996	59	84.38
*UEC*	−3.149	107%	0.999	58	85.79
*UEP*	−3.23	104%	0.999	58	86.24

**Table 2.** Candidate reference genes ranked according to their expression stability (*M* value) as determined by NormFinder.

Ranking	VS + RS	VS	RS	DS + CS	DS	CS

	Gene (*M* value)	Gene (*M* value)	Gene (*M* value)	Gene (*M* value)	Gene (*M* value)	Gene (*M* value)
1	*Actin* (0.112)	*Actin* (0.112)	*EF1α* (0.020)	*Actin* (0.139)	*Actin* (0.116)	*Actin* (0.133)
2	*EF1α* (0.194)	*GAPDH* (0.185)	*UEP* (0.110)	*GAPDH* (0.256)	*UBQ-LIKE* (0.268)	*GAPDH* (0.136)
3	*GAPDH* (0.230)	*TUB8* (0.187)	*GAPDH* (0.200)	*UBQ-LIKE* (0.349)	*GAPDH* (0.309)	*EF1α* (0.279)
4	*UEP* (0.287)	*UBQ-LIKE* (0.229)	*Actin* (0.201)	*TUB5* (0.419)	*TUB5* (0.432)	*UBQ-LIKE* (0.387)
5	*UBQ-LIKE* (0.406)	*EF1α* (0.289)	*TUB8* (0.429)	*EF1α* (0.434)	*TUB8* (0.476)	*TUB5* (0.436)
6	*TUB8* (0.424)	*TUA3* (0.364)	*UEC* (0.459)	*TUA3* (0.467)	*EF1α* (0.489)	*TUA3* (0.470)
7	*UEC* (0.486)	*UEP* (0.394)	*UBQ-LIKE* (0.509)	*TUB8* (0.497)	*TUA3* (0.549)	*TUB8* (0.475)
8	*18S* (0.646)	*TUB5* (0.416)	*TUB5* (0.597)	*UEP* (0.593)	*UEP* (0.586)	*UEC* (0.592)
9	*TUB5* (0.678)	*UEC* (0.459)	*18S* (0.654)	*UEC* (0.664)	*UBQ10* (0.662)	*UEP* (0.631)
10	*TUA3* (0.886)	*18S* (0.692)	*TUA3* (0.988)	*18S* (0.763)	*UEC* (0.686)	*18S* (0.634)
11	*UBQ10* (1.185)	*UBQ10* (0.807)	*UBQ10* (1.030)	*UBQ10* (0.848)	*18S* (0.757)	*UBQ10* (0.897)

**Table 3.** Primer sequences, PCR amplicon length of the selected reference genes and stress responsive genes in *Jatropha*.

Gene ID	Gene symbol	Primer sequences	Amplicon length
Jcr4S06558.10 a	*Actin*	F: 5′-CTCCTCTCAACCCCAAAGCCAA-3′ R: 5′-CACCAGAATCCAGCACGATACCA-3′	147 bp
Jcr4U29393.10 a	*GAPDH*	F: 5′-TGAAGGACTGGAGAGGTGGAAGAGC-3′ R: 5′-ATCAACAGTTGGAACACGGAAAGCC-3′	140 bp
Jcr4S00045.200 a	*UBQ10*	F: 5′-AAAGCAGTTGGAGGATGGAAGGAC-3′ R: 5′-GCGAAGCCTGAGAACAAGGTGAAG-3′	82 bp
Jcr4S10519.50 a	*UBQ-LIKE*	F: 5′-GGTGAGAGTGAAGTGTAATGATGACGAC-3′ R: 5′-CCTCAGAGTTATATGGTCCTTGTAAATGG-3′	136 bp
Jcr4508473.50 a	*UEP*	F: 5′-AATCCCTCCAGACCAGCAGCGACT-3′ R: 5′-GCTCTTGTAGAACTGAAGCACGGC-3′	220 bp
Jcr4S00542.10 a	*EF1α*	F: 5′-AAGATGATTCCCACCAAGCCCA-3′ R: 5′-CACAGCAAAACGACCCAGAGGA-3′	72 bp
GW880075.1 b	*TUA3*	F: 5′-TTCAATCAGCGAAAATGAGAGAGTG-3′ R: 5′-TCACTGAAAAAGGTGTTGAAGGCA-3′	178 bp
GW877086.1 b	*TUB8*	F: 5′-GCAGGGAATAACTGGGCTAAAGGT-3′ R: 5′-CTCCACCCAACGAATGACAAACTT-3′	136 bp
EZ114400.1 b	*UEC*	F: 5′-GTCCCTGATTTTGAGATGGCGTC-3′ R: 5′-CAATATGTCAAGACAAATGCTCCCG-3′	284 bp
GW878948.1 b	*TUB5*	F: 5′-TATGTTCCCAGGGCGGTTCTAATG-3′ R: 5′-GGACTGCCCAAAGACAAAGTTATCG-3′	111 bp
AY823528.1 b	*18S*	F: 5′-CTCAACCATAAACGATGCCGACC-3′ R: 5′-TTCAGCCTTGCGACCATACTCCC-3′	117 bp
Jcr4S01474.40 a	*JcRD29b*	F: 5′-AATCTCCGCAAAGAATGTTGTAGC-3′ R: 5′-CTCCCTGTCTCAGCAACTTTCTCATA-3′	180 bp
Jcr4S27135.10 a	*JcDREB1A*	F: 5′-CGGATGGACTTTTAGGGGATGAAT-3′ R: 5′-CACTGAGGTGGAGGCAACAACA-3′	160 bp

a accession number in *Jatropha* genome database [52];

b GenBank accession number; F, forward primer; R. reverse primer.
