# Supplementary material for: Selection of Reliable Reference Genes for Gene Expression Studies in the Biofuel Plant Jatropha curcas Using Real-Time Quantitative PCR
Source: Int J Mol Sci. 2013 Dec 13;14(12):24338–54. doi: 10.3390/ijms141224338 (PMC3876114; doi:10.3390/ijms141224338)
Supplement: Supplementary file 1 [file ijms-14-24338-s002.pdf]

Supplementary Information

Figure S1. Specificity of primer pairs for RT-qPCR amplification. (**a**) Dissociation curve analysis for all tested genes showing a single peak; (**b**) 1.5% agarose gel image showing specific PCR products of expected size for each reference gene tested.

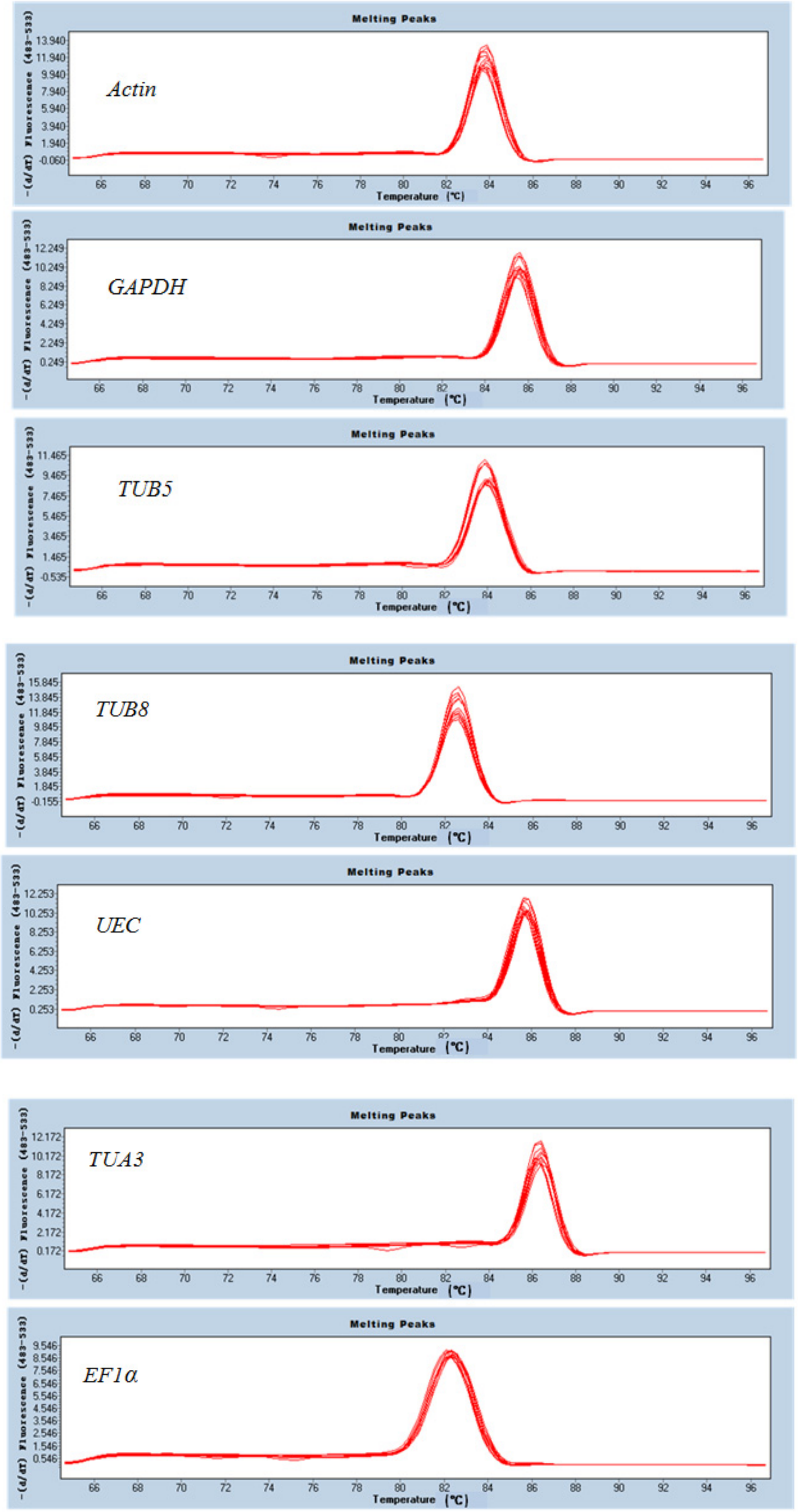

Figure S1. *Cont.*

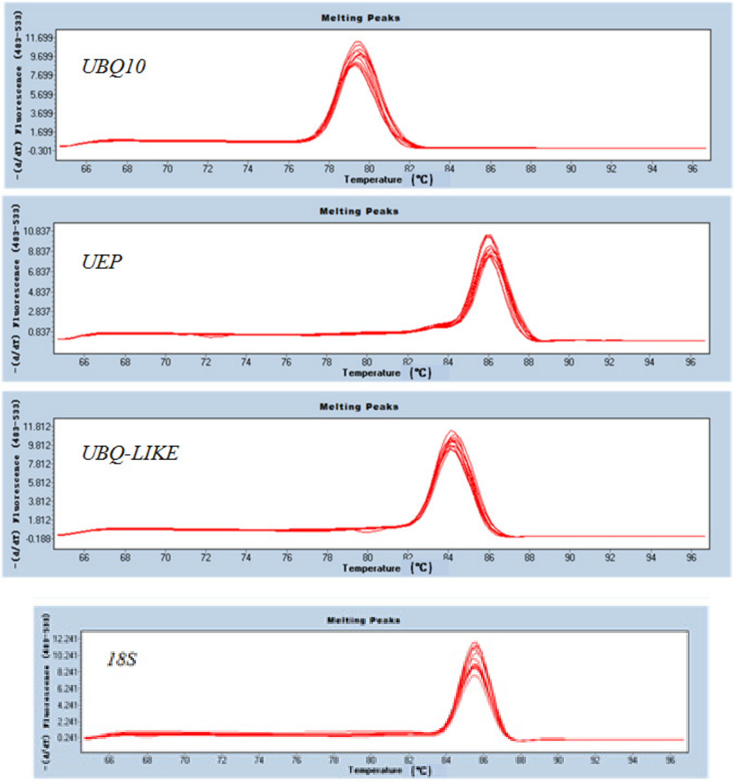

(a)

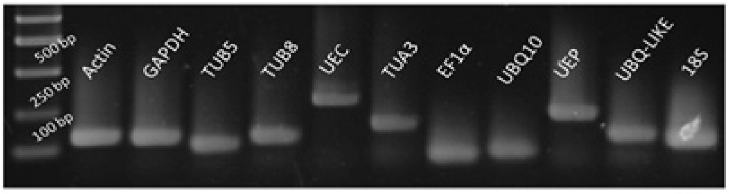

(b)

Table S1. Average C_q values of 11 candidate reference genes from various tissues in two developmental stages.

Sample	*Actin*	*GAPDH*	*TUB5*	*TUB8*	*UEC*	*TUA3*	*EF1α*	*UBQ10*	*UEP*	*UBQ-LIKE*	*18S*
Vegetative stage											
Roots	12.427	11.716	16.895	15.183	13.413	12.703	10.742	17.694	13.307	16.382	8.738
Stems	15.833	15.548	19.378	18.165	18.633	16.655	14.047	23.646	18.485	20.342	10.628
Cotyledons	15.266	15.045	19.581	17.858	16.685	16.442	13.077	22.990	16.574	18.720	10.242
Young leaves	12.729	12.404	16.906	15.534	15.509	12.829	10.796	20.839	14.652	17.388	8.928
Mature leaves	16.271	16.215	20.743	18.720	17.664	16.636	13.484	24.275	17.261	20.338	10.414
Reproductive stage											
Roots	12.882	12.313	18.448	16.430	13.780	14.549	10.912	16.182	13.209	16.361	7.941
Stems	15.594	14.868	20.272	18.816	17.505	17.813	13.860	21.659	16.949	19.340	10.925
Young leaves	11.644	10.586	17.016	14.621	11.949	12.584	9.791	15.678	12.623	15.344	7.548
Mature leaves	13.794	13.496	20.879	18.483	14.682	18.501	12.833	19.461	15.532	16.799	8.583
Flower Shoots	10.984	10.152	15.934	13.791	12.513	11.415	8.878	16.561	11.990	14.546	7.429
Seeds (20 DAP)	11.732	10.637	16.386	14.439	13.833	12.662	9.853	19.541	12.919	16.569	8.280
Average C_q	13.560	12.998	18.403	16.549	15.106	14.799	11.661	19.866	14.864	17.466	9.059
S.D.	1.889	2.162	1.845	1.913	2.247	2.481	1.841	3.077	2.210	1.957	1.278

Table S2. Average C_q values of 11 candidate reference genes from various tissues in desiccation and cold stress treatments.

**Sample**	***Actin***	***GAPDH***	***TUB5***	***TUB8***	***UEC***	***TUA3***	***EF1α***	***UBQ10***	***UEP***	***UBQ-LIKE***	***18S***
Roots (Control)	13.183	12.376	17.464	16.518	13.661	13.718	12.648	19.393	13.360	14.604	8.344
hypocotyls (Control)	16.390	15.474	19.685	18.980	18.229	16.509	16.183	23.453	17.548	18.577	10.157
young leaves (Control)	13.521	12.926	16.841	17.103	14.954	13.506	12.753	19.630	14.067	14.774	8.152
Roots (DS3)	13.649	12.730	18.380	18.162	14.276	14.763	12.379	18.542	13.379	14.722	8.560
hypocotyls (DS6)	17.648	16.042	22.059	21.540	20.731	19.605	15.619	23.261	19.309	19.672	10.352
young leaves (DS12)	13.564	12.710	17.342	16.620	14.023	13.649	12.454	20.385	12.747	14.406	6.905
Roots (CS6)	13.094	11.779	17.341	16.662	13.742	13.104	12.512	16.987	13.066	14.475	8.815
hypocotyls (CS12)	16.198	14.716	19.270	18.440	18.912	17.660	14.645	20.482	18.090	18.854	9.908
young leaves (CS24)	11.801	11.397	16.512	16.049	13.923	12.671	11.573	16.848	12.328	13.934	7.152
Average C_q	14.339	13.350	18.322	17.786	15.828	15.020	13.419	19.887	14.877	16.002	8.705
S.D.	1.926	1.652	1.763	1.725	2.702	2.380	1.630	2.358	2.660	2.305	1.244

"Control", represents the samples do not treated by stress; "DS", desiccation stress; "CS", cold stress in 4 °C; Numbers after the DS or CS represent hours for treatments.

Table S3. Sequences of the cDNA fragments of eleven candidate reference genes for RT-qPCR used in this study.

Gene	Sequences of the cDNA fragments
Actin	CTCCTCTCAACCCCAAAGCCAACAGAGAAAAGATGACCCAAATCATGTTTG AGACATTTAATGTGCCAGCAATGTATGTCGCCATCCAGGCTGTTCTGTCTTTG TATGCCAGTGGTCGTACAACTGGTATCGTGCTGGATTCTGGTG (147 bp)
GAPDH	TGAAGGACTGGAGAGGTGGAAGAGCTGCCTCCTTCAACATCATTCCTAGCA GCACTGGAGCTGCTAAGGCTGTTGGGAAGGTGCTACCTGCTCTGAACGGCA AGCTTACTGGAATGGCTTTCCGTGTTCCAACTGTTGAT (140 bp)
TUB5	TATGTTCCCAGGGCGGTTCTAATGGATCTTGAGCCTGGCACCATGGACAGTAT CAGATCCGGTCCTTATGGACAGATCTTCAAGCCCGATAACTTTGTCTTTGGGC AGTCC (111 bp)
TUB8	CAGGGAATAACTGGGCTAAAGGTCATTACACTGAAGGAGCTGAGTTGATCG ATTCTGTGCTTGATGTTGTCAGGAAAGAGGCTGAGAATTGTGATTGCTTGCA GGGATTTCAAGTTTGTCATTCGTTGGGTGGAG (135 bp)
UEC	GTCCCTGATT TTGAGATGGC GTCGAAGCGG ATCTTGAAGG AACTCAAAGGATTTACAGAA GGATCCTCCC ACATCATGCA GCGCTGGTCC CGTTGCTTGAAGACATGTTT CATTGGCAAG CTACGATCAT GGGTCCCCCT GACAGTCCATATGCAGGGGG TGTTTTCCTT GTTACTATTC ATTTCCCTCC GGATTATCCATTTAAGCCTC CCAAGGTGGC TTTCAGAACA AAGGTATTCC ACCCAAATATTAACAGCAAC GGGAGCATTT GTCTTGACAT ATTG (284 bp)
TUA3	TTCAATCAGCGAAAATGAGAGAGTGCATTTCAATCCACATTGGTCAGGCTGG TATTCAGGTTGGAAATGCCTGCTGGGAGCTTTACTGTCTTGAGCATGGCATTC AGCCTGATGGCCAGATGCCAAGTGACAAGACCGTCGGTGGAGGTGACGATG CCTTCAACACCTTTTTCAGTGA (178 bp)
EF1α	AAGATGATTCCCACCAAGCCCATGGTTGTGGAGACCTTTTCTGAGTACCCTC CTCTGGGTCGTTTTGCTGTG (72 bp)
UBQ10	AAAGCAGTTGGAGGATGGAAGGACTCTTGCTGATTACAATATCCAAAAGGA ATCTACTCTTCACCTTGTTCTCAGGCTTCGC (82 bp)
UEP	AATCCCTCCAGACCAGCAGCGACTGATCTTCGCCGGAAAGCAGCTAGAGGA TGGCCGCACTCTTGCCGACTACAACATCCAGAAGGAATCCACCCTCCACCTG GTGCTTCGTCTTCGTGGTGGTGCTAAGAAGAGAAAGAAGAAGACTTACACC AAGCCAAAGAAGATCAAGCACAAGAAGAAGAAGGTCAAACTTGCCGTGCT TCAGTTCTACAAGAGC (220 bp)
UBQ-LIKE	GGTGAGAGTGAAGTGTAATGATGACGACACCATTGGCGATCTGAAAAAACT GGTGGCGGCGCAGACCGGTACCAGAGCTGAGAAGATAAGGATACAGAAGT GGTACACCATTTACAAGGACCATATAACTCTGAGG (136 bp)
18S rRNA	CTCAACCATAAACGATGCCGACCAGGGATCGGCGGATGTTGCTTTTAGGACT CCGCCGGCACCTTATGAGAAATCAAAGTCTTTGGGTTCCGGGGGGAGTATGG TCGCAA GGCTGAAA (118 bp)

Figure S2. The expression level of *Actin* (**a**), *GAPDH* (**b**) and *TUB8* (**c**) in different cDNA pools that were reverse transcribed using gene-specific primer mix and Oligo-d (T) primer.

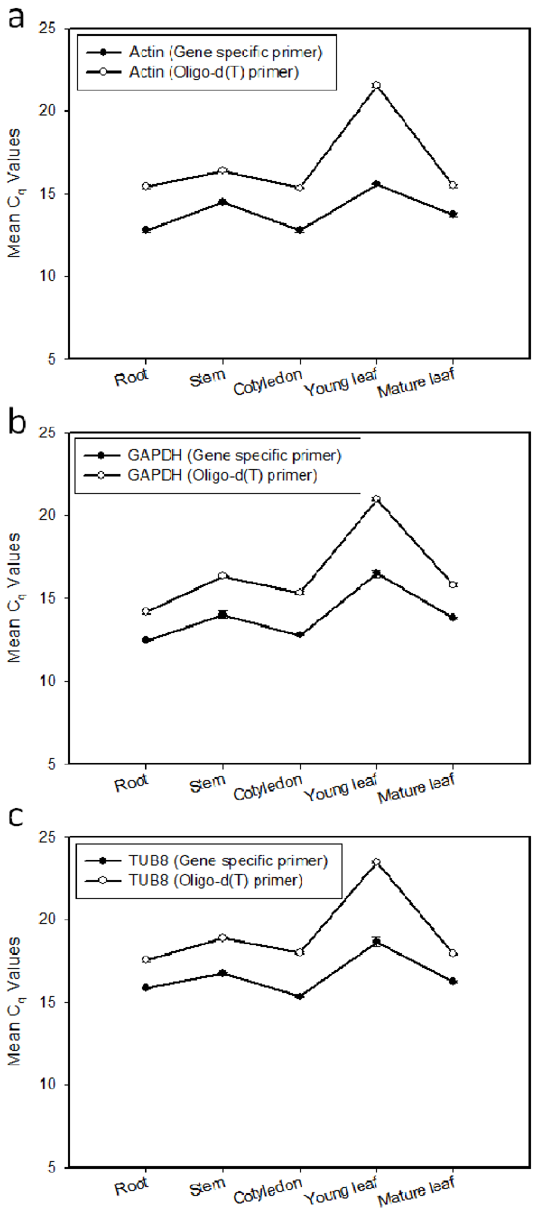

© 2013 by the authors; licensee MDPI, Basel, Switzerland. This article is an open access article distributed under the terms and conditions of the Creative Commons Attribution license (http://creativecommons.org/licenses/by/3.0/).
